# COVID-19: age, Interleukin-6, C-reactive protein, and lymphocytes as key clues from a multicentre retrospective study

**DOI:** 10.1186/s12979-020-00194-w

**Published:** 2020-08-14

**Authors:** Aurora Jurado, María C. Martín, Cristina Abad-Molina, Antonio Orduña, Alba Martínez, Esther Ocaña, Oscar Yarce, Ana M. Navas, Antonio Trujillo, Luis Fernández, Esther Vergara, Beatriz Rodríguez, Bibiana Quirant, Eva Martínez-Cáceres, Manuel Hernández, Janire Perurena-Prieto, Juana Gil, Sergi Cantenys, Gema González-Martínez, María T. Martínez-Saavedra, Ricardo Rojo, Francisco M. Marco, Sergio Mora, Jesús Ontañón, Marcos López-Hoyos, Gonzalo Ocejo-Vinyals, Josefa Melero, Marta Aguilar, Delia Almeida, Silvia Medina, María C. Vegas, Yesenia Jiménez, Álvaro Prada, David Monzón, Francisco Boix, Vanesa Cunill, Juan Molina

**Affiliations:** 1 Department of Immunology and Allergology, Hospital Universitario Reina Sofía-Instituto de Investigación Biomédica de Córdoba (IMIBIC), Córdoba, Spain; 2 Centro de Hemoterapia y Hemodonación de Castilla y León, Paseo de Filipinos s/n, 47007 Valladolid, Spain; 3 Department of Microbiology and Immunology, Hospital Clínico Universitario, Valladolid, Spain; 4 Laboratory Unit. Complejo Hospitalario, Jaén, Spain; 5 Laboratoy of Immunology and Genetics. Hospital San Pedro de Alcántara, Cáceres, Spain; 6 Laboratory Unit. Hospital Juan Ramón Jiménez, Huelva, Spain; 7 Department of Immunology, Hospital Germans Trias i Pujols, Barcelona, Spain; 8 Department of Immunology, Hospital Universitario Vall d’Hebron, Barcelona, Spain; 9 Department of Immunology, Hospital General Universitario e Instituto de Investigación Sanitaria “Gregorio Marañón”, Madrid, Spain; 10 Unit of Immunology, Hospital Universitario Insular-Materno Infantil, Las Palmas de Gran Canaria, Spain; 11 Department of Immunology, Complejo Hospitalario, La Coruña, Spain; 12 Laboratory Unit. Hospital General, Alicante, Spain; 13 Laboratory Unit. Hospital General Universitario, Albacete, Spain; 14 Department of Immunology, Hospital Universitario Marqués de Valdecilla, Santander, Spain; 15 Department of Immunology, Hospital Infanta Cristina, Badajoz, Spain; 16 Laboratory Unit. Complejo Hospitalario Nuestra Señora de la Candelaria, Santa Cruz de Tenerife, Spain; 17 Department of Immunology, Fundación Jiménez Díaz, Madrid, Spain; 18 Department of Immunology, Hospital de Donostia, San Sebastián, Spain; 19 Department of Immunology, Hospital Clínico Universitario, Salamanca, Spain; 20 Department of Immunology, Hospital Universitario Son Espases, Palma de Mallorca, Spain

**Keywords:** Severe acute respiratory syndrome coronavirus 2, COVID-19, Immunosenescence, Immunity, Renin-angiotensin system, ACE2, Interleukin-6, C-reactive protein, Lymphocytes, Spain

## Abstract

**Background:**

The SARS-CoV-2 infection has widely spread to become the greatest public health challenge to date, the COVID-19 pandemic. Different fatality rates among countries are probably due to non-standardized records being carried out by local health authorities. The Spanish case-fatality rate is 11.22%, far higher than those reported in Asia or by other European countries. A multicentre retrospective study of demographic, clinical, laboratory and immunological features of 584 Spanish COVID-19 hospitalized patients and their outcomes was performed. The use of renin-angiotensin system blockers was also analysed as a risk factor.

**Results:**

In this study, 27.4% of cases presented a mild course, 42.1% a moderate one and for 30.5% of cases, the course was severe. Ages ranged from 18 to 98 (average 63). Almost 60 % (59.8%) of patients were male. Interleukin 6 was higher as severity increased. On the other hand, CD8 lymphocyte count was significantly lower as severity grew and subpopulations CD4, CD8, CD19, and NK showed concordant lowering trends. Severity-related natural killer percent descents were evidenced just within aged cases. A significant severity-related decrease of CD4 lymphocytes was found in males. The use of angiotensin-converting enzyme inhibitors was associated with a better prognosis. The angiotensin II receptor blocker use was associated with a more severe course.

**Conclusions:**

Age and age-related comorbidities, such as dyslipidaemia, hypertension or diabetes, determined more frequent severe forms of the disease in this study than in previous literature cohorts. Our cases are older than those so far reported and the clinical course of the disease is found to be impaired by age. Immunosenescence might be therefore a suitable explanation for the hampering of immune system effectors. The adaptive immunity would become exhausted and a strong but ineffective and almost deleterious innate response would account for COVID-19 severity. Angiotensin-converting enzyme inhibitors used by hypertensive patients have a protective effect in regards to COVID-19 severity in our series. Conversely, patients on angiotensin II receptor blockers showed a severer disease.

## Background

SARS-CoV-2 infection has become widespread. Never before have we experienced a health emergency like this. At the time of writing, 6 months after the first diagnosed case [1] the virus has infected 12.270.172 people, with an overall case-fatality rate of 4.52% [2] far exceeding the 1% reported outside the epicentre by early studies [3]. It can be traced back to the end of February, when the pandemic started to rapidly expand, hitting some European countries the hardest, such as Spain, with case-fatality rates around 11.22%. We lack so far, an explanation to such big differences. They might be related to different local approaches for records and statistics of infected cases in each country. Absolute mortality rates are far higher in Spain than those reported in Asia or other European countries [4].

In 2002, during the SARS-CoV epidemic, a coronavirus was for the first time revealed to be highly pathogenic. Coronaviruses were until then considered to cause just mild infections, mainly in immunocompromised people [5]. SARS-CoV-2 has shown much higher infectivity than SARS-CoV, with a doubling time of 2.3–3.3 days, and a basic reproductive number (R_0_) of 5.7 [6]. SARS-CoV-2 can be considered especially challenging due to its several intrinsic and extrinsic characteristics. It has a highly variable prevalence and outcomes within countries depending on age, weather, and social habits.

The angiotensin-converting enzyme 2 (ACE2) is the receptor for SARS-CoV-2 and plays a key role in human infection [7]. The ACE2 has two isoforms; a large one anchored to the cell membrane [8] and a small soluble isoform lacking anchorage to the membrane and circulates at low concentrations in blood [9]. It has been therefore suggested that the use of drugs increasing ACE2 expression, such as angiotensin-converting enzyme inhibitors (ACEI) and angiotensin II receptor blockers (ARB), could enhance infection [10]. On the other hand, increasing soluble ACE2 may be a therapeutic tool to competitively inhibit the virus [11]. Smoking can cause an increase in ACE2 expression and might, therefore, be a risk factor for SARS CoV2 infection [12].

Both innate and adaptive responses are involved in fighting against SARS-CoV-2 [13]. An accurate immune response is essential for infection resolution. An aberrant immune response might be the key to understanding the immunopathogenesis of SARS-CoV-2 infection. It seems that the progression to severe COVID-19 could be associated with a poor adaptive immune response [14] and with an innate immune response exacerbation, with an increase in plasma levels of both cytokines and pro-inflammatory chemokines [15].

Understanding the pathogenesis of the virus as well as identifying risk or severity factors for COVID-19, are key points for identifying disease evolution biomarkers, and taking immediate preventive actions.

This study aimed to obtain, within the shortest possible time, a reliable snapshot of the demographic and clinical characteristics of COVID-19 patients admitted to Spanish hospitals during the first month of the pandemic and to reveal severity risk factors. This knowledge would help manage both clinical and health decisions.

## Results

### Baseline demographic characteristics, risk factors, and COVID-19 therapies

A total of 584 SARS-CoV-2 infected inpatients from 19 Spanish Hospitals were included. Twenty-seven percent (27.4%) of cases presented a mild disease, 42.1% a moderate one, and 30.5% a severe one. By data collection deadline, 278 patients have been discharged and 87 have died. The descriptive baseline characteristics of the population (valid n, frequencies, percentages, mean, median, standard deviation, and interquartile range) are shown in Table 1. Categorical variables stratified by severity are shown in Table 2.

**Table 1:** Baseline characteristics of the study population

Clinical and demographic characteristics	All patients *n* = 584; (%)					
Severity						
Mild	160 (27.4)					
Moderate	246 (42.1)					
Severe	178 (30.5)					
Gender						
Male	349 (59.8)					
Female	235 (40.2)					
Hypertension	293 (52.0)					
RASB^a^ intake						
no	56 (21.1)					
yes	209 (78.9)					
Dyslipidemia	159 (28.8)					
Diabetes	131 (23.7)					
Immunodeficiency (primary or secundary)	40 (6.8)					
	Ref.v^b^	n	Mean	Median	SD^c^	IQR^d^
Age		584	63.0	64.0	16.5	52–76
**laboratory data on admission**						
IL6^e^ (pg/mL)	< 4.4	254	113.7	41.0	355.2	15.3–94.6
CRP^f^ (mg/L)	< 10	523	111.30	87.00	93.70	39–153.2
ferritin (ng/mL)	20–250	297	1108.60	793.00	1524.30	361–1417
D-dimer (ng/mL)	< 500	456	1885.10	620.00	8214.20	399–1169
LDH^g^ (U/L)	120–246	467	334.10	291.00	186.90	232–394
days from onset to admission		548	7.20	7.00	5.10	4–10
Leucocyte count (cells*10^3^/μL)	4–12.4	570	7.57	6.39	5.60	4.82–9.00
Neutrophil count (cells*10^3^/μL)	1.9–8	570	5.65	4.62	3.70	3.30–7.12
Lymphocyte count (cells*10^3^/μL)	0.9–5	570	1.16	0.99	1.06	0.71–1.39
Lymphocyte %	19–48	570	18.20	16.02	11.40	9.7–23.5
CD3 + CD4+ %	25–65	55	44.10	44.80	11.60	37–51.3
CD3 + CD4+ count (cells*10^3^/μL)	0.5–1.4	54	0.54	0.43	0.38	0.26–0.69
CD3 + CD8+ %	12–40	55	23.36	24.40	9.82	15.6–30.5
CD4 + CD8+ count (cells*10^3^/μL)	0.25–1	54	0.28	0.20	0.22	0.12–0.36
CD19+ %	5–20	52	12.90	11.55	73.00	8.2–15.9
CD19+ count (cells*10^3^/μL)	0.1–0.5	51	0.13	0.10	0.09	0.06–0.20
Natural Killer %	5–20	52	15.90	15.15	8.70	8.66–20.65
Natural Killer count (cells*10^3^/μL)	0.5–5	51	0.17	0.14	0.12	0.08–0.20
Immunoglobulin G	650–1600	19	961.6	933.0	131.3	885–1006
Immunoglobulin A	40–350	19	230.9	223.0	72.3	178–248
Immunoglobulin M	50–300	19	103.1	90.0	39.8	72–129
**Laboratory data at discharge**						
IL6 (pg/mL)	< 4.4	117	99.56	9	711.81	3.9–23.2
CRP (mg/L)	< 10	297	29.96	13.00	44.90	4.7–36
ferritin (ng/mL)	20–250	209	1263.56	633	6518.34	321–1137
D- dimer (μg/L)	< 500	271	3246.00	591	33,491.34	360–1149
LDH (U/L)	120–246	273	342.54	234	1142.00	195–290
days from admission to discharge		146	11.75	11	6.97	7–15
Leucocyte count (cells*10^3^/μL)	4–12.4	326	7.42	6.4	3.88	4.01–8.40
Neutrophil count (cells*10^3^/μL)	1.9–8	326	5.17	4.1	3.79	2.98–6.00
Lymphocyte count (cells*10^3^/μL)	0.9–5	326	1.51	1.44	0.76	1–1.9
Lymphocyte %	19–48	326	23.43	23.75	11.77	14.6–31.1
CD3 + CD4+ %	25–65	14	48.01	53.5	15.71	49–58.24
CD3 + CD8+ %	12–40	14	19.67	18.5	10.04	10–29.27
CD19%	5–20	14	16.97	10.93	20.32	7.9–17
Natural Killers %	5–20	14	13.02	12	7.53	sep-17

*Abbreviations*: *RASB*^a^ Renin-angiotensin system blockers, *Ref.v*^b^ Reference values, *SD*^c^ Standard deviation, *IQR*^d^ interquartile range, *IL6*^e^ Interleukin 6, *CRP*^f^ C-reactive protein, *LDH*^g^ Lactate dehydrogenase

**Table 2:** Age, gender, comorbidities and RASB intake relationship with COVID-19 severity

	Mild	Moderate	Severe
**Age (*p*** **= 0.019)**	n (%)	n (%)	n (%)
< 30	10 (43.5)	9 (39.1)	4 (17.4)
30–45	26 (37.7)	26 (37.7)	17 (24.6)
45–60	41 (26.6)	62 (40.3)	51 (33.1)
60–75	57 (30.6)	80 (43.0)	49 (26.3)
> 75	26 (17.1)	69 (45.4)	57 (37.5)
**Gender (*p*** **< 0.001)**			
Male	77 (22.1)	144 (41.3)	128 (36.7)
Female	83 (35.3)	102 (43.4)	50 (21.3)
**Hypertension (*p*** **= 0.015)**			
No	91 (33.7)	107 (39.6)	72 (26.7)
Yes	67 (22.9)	132 (45.1)	94 (32.1)
**Dyslipidemia (*p*** **= 0.006)**			
No	127 (32.2)	159 (40.4)	108 (27.4)
Yes	30 (18.9)	75 (47.2)	54 (34.0)
**Diabetes (*p*** **= 0.003)**			
No	134 (31.8)	175 (41.5)	113 (26.8)
Yes	23 (17.6)	59 (45.0)	49 (37.4)
**Immunodeficiency**			
No	283 (60.6)	379 (81.0)	273 (58.4)
Yes	8 (68.4)	21 (102.6)	11 (28.9)
**RASB**^**a**^ **intake**			
No	10 (17.9)	32 (57.1)	14 (25.0)
Yes	50 (23.9)	91 (43.5)	68 (34.0)
	**Mild-Moderate**	**Severe**	
**RASB**^**a**^ **intake**	n (%)	n (%)	
No	42 (75)	14 (25)	
Yes	142 (67.9)	67 (32.1)	
**ACE**^**b**^ **intake (*p*** **= 0.046)**			
No	111 (65.3)	59 (34.7)	
Yes	71 (77.2)	21 (22.8)	
**ARB**^**c**^ **intake (*p*** **= 0.004)**			
No	95 (77.9)	27 (22.1)	
Yes	76 (60.8)	49 (39.2)	

*Abbreviations*: *p* Chi Squared p-values, *RASB*^a^ Renin-angiotensin system blockers, *ACE*^b^ Angiotensin-converting enzyme inhibitors, *ARB*^c^ Angiotensin II receptor blockers

Almost 60 % (59.8%) of the cases were male. Ages in our cohort ranged from 18 to 98 years old, 63 years old as an average (SD 16.5). Concerning comorbidities, 52.0% were hypertensive, 78.9% of them were treated with blockers of the renin-angiotensin system (RASBs); 28 % 28.8% had dyslipidaemia and 23.7% suffered diabetes. Immunodeficiency was most often secondary to other processes, such as transplantation or chemotherapy treatment. These cases accounted for 6.8% (*n* = 40) as seen in Table 1.

**Fig. 1:**
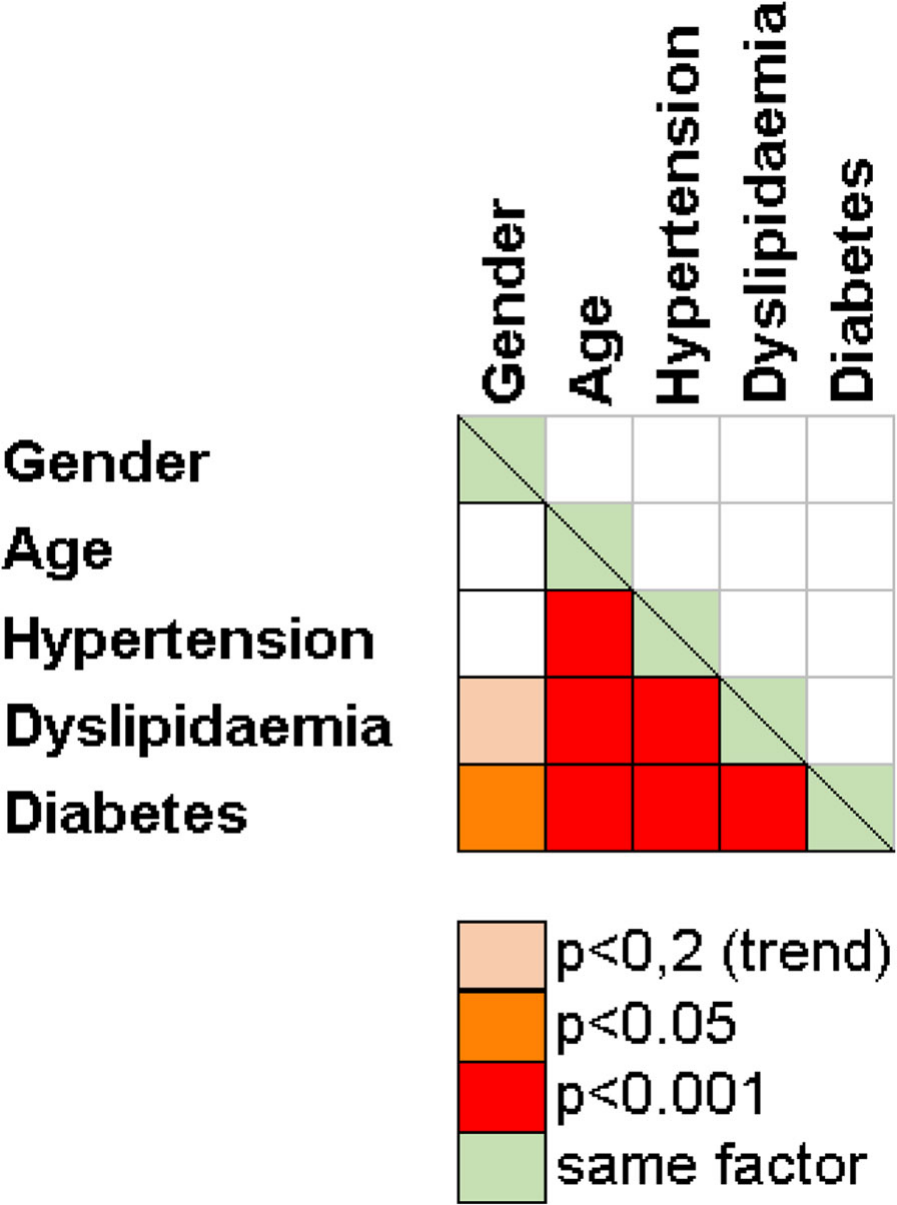
Severity factors and comorbidities interactions. Pearson’s Chi Squared p-values

Hypertension, dyslipidaemia, and diabetes become more frequent with age (*p* < 0.001), (Table 3). These four risk factors showed strong interference (Fig. 1). Nevertheless, a predictive model could not be proposed due to frequent missing values.

**Table 3:** Influence of age and gender on comorbidities

				Age			Gender	
		< 30	30–45	45–60	60–75	> 75	Male	Female
		n (%)	n (%)	n (%)	n (%)	n (%)	n (%)	n (%)
**Hypertension**^a^	no	21 (7.8)	55 (20.4)	97 (35.9)	65 (24.1)	32 (11.9)	155 (57.4)	115 (42.6)
	yes	1 (0.3)	9 (3.1)	50 (17.1)	116 (39.6)	117 (39.9)	182 (62.1)	111 (37.9)
**Dyslipidaemia**^a^	no	22 (5.6)	59 (15.0)	117 (29.7)	108 (27.4)	88 (22.3)	227 (57.6)	167 (42.4)
	yes	0 (0.0)	3 (1.9)	30 (18.9)	68 (42.8)	58 (36.5)	103 (64.8)	56 (35.2)
**Diabetes**^a^	no	21 (5.0)	58 (13.7)	128 (30.3)	114 (27.0)	101 (23.9)	241 (57.1)	181 (42.9)
	yes	1 (0.8)	6 (4.6)	19 (14.5)	63 (48.1)	42 (32.1)	88 67.2)	43 (32.8)

^a^all Chi Squared p-values either vs age or gender were < 0.001

Moderate and severe forms were found to be significantly associated with older age, specially over 75 (*p* = 0.019; OR = 2.179 (1.363–3.482)), male gender (*p* < 0.001; OR = 1.929(1.334–2.788)), dyslipidaemia (*p* = 0.006; OR = 2.045 (1.304–3.208)), hypertension (*p* = 0.015; OR = 1.715(1.182–2.486)) and diabetes (*p* = 0.003; OR = 2.184(1.332–3.583)). Severe cases over the age of 75 accounted for 37.5%. The use of renin-angiotensin system blockers (RASB) by hypertensive patients revealed no difference regarding mild, moderate, or severe forms of the disease. However, differences arose when considering patients who developed a more serious picture compared to those who had a mild-moderate course. Intake of RASB showed again no effect regarding COVID-19 severity. Meanwhile, when assessing the use of single RASBs, the intake of ACEI was associated with a better prognosis ((*p* = 0.046; Odds Ratio for severe COVID-19 was 0.56 with a 95% Confidence Interval (0.31–0.99)). On the contrary, the use of ARB was related to higher severity (p 0.004; Odds Ratio for severe COVID-19 was 2.26 with 95% Confidence Interval (1.29–3.96)) (Table 2).

Once at hospital, 84.2% of inpatients received antibiotics; the most commonly prescribed ones were azithromycin combinations (71.3%); those treated with antimalarial drugs accounted for 71.7 and 65.8% received antivirals, being lopinavir/ritonavir being the most widely used. Around one-half of cases (50.2%), received combined therapy consisting of antibiotics, antimalarials, and antivirals (commonly named triple therapy). Immunosuppressant drugs were used in 18.3% of cases. Anti-cytokine therapy was used in 8.4%, mostly anti-IL-6R (Tocilizumab), and 17.3% were treated with either α or β interferon.

### Laboratory parameters on admission and at discharge

On admission, means of laboratory parameters, IL-6, CRP, ferritin, D-dimer, LDH, leukocyte, and neutrophil counts, were above usual reference ranges (those ranges can slightly change within centres), in contrast to lymphocyte counts and percentages as well as lymphocyte subset counts, that are within the lower part of their ranges (Table 1). Higher severity was significantly associated with higher levels of IL-6, CRP, ferritin, D-dimer, LDH, leukocyte, and neutrophil counts, but with lower lymphocyte percentages and counts (Table 4). The mean percentages of lymphocyte subpopulations (*n* = 54) were within normal ranges. CD8 Lymphocyte count was found to be significantly higher in mild cases, similar trends were found for CD4, CD19, and NK cell counts. IgG and IgM values were as well inversely related to severity (Table 4).

**Table 4:** Age and Laboratory results. Association to COVID-19 severity and evolution from admission to discharge

	Severity *p*-value	Δa-d^a^ p-value	n	mean	median	SD^b^	IQR^c^
**Age**	< 0.001(A)						
Mild			160	58.96	61.00	17.08	49–70.5
Moderate			246	64.08	66.00	16.05	54–77
Severe			178	65.25	65.50	15.98	54–79
***On admission***							
**IL6**^**d**^ **(pg/mL)**	< 0.001						
Mild			78	31.40	17.60	40.53	9–40.9
Moderate			98	77.86	43.10	155.30	19.5–87.3
Severe			78	241.16	87.45	597.92	30.4–239.7
**CRP**^**e**^ **(mg/L)**	< 0.001						
Mild			132	66.21	44.45	67.83	17.45–85.2
Moderate			231	108.82	93.00	83.25	43.8–147
Severe			160	152.14	128.40	107.91	64.25–217.65
**ferritin (ng/mL)**	< 0.001						
Mild			80	711.39	491.45	881.96	201.65–874
Moderate			133	1003.30	775.00	902.63	390–1479
Severe			84	1653.65	1073.50	2404.04	713.5–1796.5
**D-dimer (ng/mL)**	< 0.001						
Mild			120	1083.58	522.00	3684.33	340.5–797
Moderate			200	1442.13	594.00	3878.02	391.5–1025
Severe			136	3243.72	960.50	13,804.06	468.5–1586
**LDH**^**f**^ **(U/L)**	< 0.001						
Mild			124	252.55	244.50	75.01	200–292.5
Moderate			208	314.61	292.50	123.08	240–372.5
Severe			135	438.99	401.00	274.08	279–524
Leucocyte count (cells*10^**3**^**/μL)**	< 0.001						
Mild			147	6.25	5.70	2.46	4.68–7.03
Moderate			246	7.60	6.15	7.16	4.70–8.82
Severe			177	8.65	7.89	4.73	5.50–10.3
Neutrophil count (cells*10^**3**^**/μL)**	< 0.001						
Mild			147	4.33	3.89	2.22	3.00–5.17
Moderate			246	5.39	4.32	3.74	3.20–7.00
Severe			177	7.10	6.40	4.13	4.30–8.71
Lymphocyte count (cells*10^**3**^**/**μL**)**	0.048						
Mild			147	1.29	1.10	0.76	0.86–1.57
Moderate			246	1.13	0.97	0.85	0.73–1.33
Severe			177	1.10	0.90	1.47	0.59–1.24
**Lymphocyte %**	< 0.001						
Mild			147	22.04	19.70	11.14	14.7–28.7
Moderate			246	18.55	17.10	10.99	9.8–24.5
Severe			177	14.64	12.00	11.01	7.5–18.1
**CD3 + CD4+ %**							
Mild			8	41.31	41.10	6.56	36–47.1
Moderate			35	45.84	46.50	12.52	38.3–52.8
Severe			12	41.04	41.90	11.25	34.3–50.4
CD3 + CD4+ count (cells*10^**3**^**/μL)**							
Mild			8	0.74	0.71	0.46	0.32.35–1.1
Moderate			33	0.56	0.44	0.39	0.27–0.69
Severe			13	0.36	0.32	0.20	0.27–0.46
**CD3 + CD8+ %**							
Mild			8	26.23	27.00	4.26	22.7–28.9
Moderate			35	21.74	20.30	10.66	12.09–30.5
Severe			12	26.57	28.30	9.18	19.39–32.4
CD4 + CD8+ count (cells*10^**3**^**/μL)**	0.041(A)						
Mild			8	0.45	0.41	0.28	0.20–0.70
Moderate			33	0.25	0.18	0.20	0.13–0.35
Severe			13	0.24	0.24	0.18	0.084–0.30
**CD19+ %**							
Mild			8	11.50	10.90	3.36	8.95–13
Moderate			35	12.47	10.60	6.99	7.3–16
Severe			9	15.97	12.00	10.61	10.3–14.83
CD19+ count (cells*10^**3**^**/μL)**							
Mild			8	0.19	0.18	0.11	0.09–0.29
Moderate			33	0.12	0.09	0.09	0.06–0.20
Severe			10	0.16	0.10	0.08	0.06–0.12
**Natural Killer %**							
Mild			8	15.59	13.80	8.96	8.55–23.55
Moderate			35	16.06	15.50	8.36	11.8–20.7
Severe			9	15.67	11.40	10.90	6.8–20.6
Natural Killer count (cells*10^**3**^**/μL)**							
Mild			8	0.23	0.16	0.15	0.12–0.37
Moderate			33	0.17	0.16	0.12	0.08–0.21
Severe			10	0.11	0.12	0.06	0.09–0.14
**IgG (mg/dL)**	0.048						
Mild			1	1006.00	1006.00	.	1006–1006
Moderate			13	998.31	934.00	133.23	915–1071
Severe			5	857.20	862.00	76.43	788–885
**IgA (mg/dL)**							
Mild			1	248.00	248.00	.	248–248
Moderate			13	234.00	223.00	86.48	175–248
Severe			5	219.40	218.00	28.98	213–230
**IgM (mg/dL)**	0.009						
Mild			1	129.00	129.00	.	129–129
Moderate			13	118.00	121.00	34.88	88–141
Severe			5	59.20	58.00	13.83	50–72
***At discharge***							
**IL6 (pg/mL)**	0.017						
Mild			55	21.17	11.60	28.51	4.77–23.2
Moderate		< 0.001	50	47.04	7.26	126.46	1.88–13.4
Severe			12	677.75	24.86	2204.56	9.1–59.63
**CRP (mg/L)**		< 0.001					
Mild			115	27.69	14.30	32.92	6.3–38.1
Moderate			137	29.93	13.93	42.69	4–36
Severe			45	35.86	8.00	71.26	4–25.2
**ferritin (ng/mL)**	< 0.001						
Mild			77	611.96	386.00	646.97	245–793
Moderate			94	778.67	687.50	599.84	331–1178
Severe			38	3783.41	1085.90	15,135.72	571–1776
**D-dimer (ng/mL)**	< 0.001						
Mild			94	705.22	463.50	937.24	326–751
Moderate			130	5219.08	586.00	48,290.67	356–1040
Severe		< 0.001	47	2870.13	1415.00	4230.08	792–3912
**LDH (U/L)**	< 0.001	0.004					
Mild			102	238.22	218.00	78.79	192–261
Moderate			124	247.19	235.50	83.32	192–271
Severe			47	820.53	286.00	2722.15	242–400
Leucocyte count (cells*10^**3**^**/μL)**	< 0.001	0.013					
Mild			127	6.26	5.80	2.59	4.78–7.21
Moderate			146	7.46	6.90	3.08	5.30–9.20
Severe			53	10.10	8.31	6.41	5.79–12.33
Neutrophil count (cells*10^**3**^**/**μL**)**	< 0.001						
Mild		< 0.001	127	4.12	3.54	2.56	2.67–4.50
Moderate			146	5.07	4.40	2.99	3.09–6.20
Severe			53	8.01	6.50	6.18	3.78–9.60
Lymphocyte count (cells*10^**3**^**/μL)**		< 0.001					
Mild			127	1.50	1.45	0.62	1.06–1.89
Moderate			146	1.54	1.40	0.87	0.97–1.87
Severe			53	1.52	1.54	0.77	0.8–2.02
**Lymphocyte %**	0.006	0.007					
Mild			127	25.47	26.10	9.31	19.4–32.1
Moderate			146	23.12	22.65	12.73	13.7–30.3
Severe			53	19.41	18.40	13.34	8.6–26.6
**CD3 + CD4+ %**							
Mild			3	49.33	54.00	13.61	34–60
Moderate			11	47.66	53.00	16.84	49–58.24
Severe			0				
**CD3 + CD8+ %**							
Mild			3	20.33	20.00	11.50	9–32
Moderate			11	19.50	17.00	10.22	10–29.27
Severe			0				
**CD19%**							
Mild			3	15.00	17.00	6.24	8–20
Moderate			11	17.51	10.86	22.97	7–15
Severe			0				
**Natural Killer %**							
Mild			3	13.67	15.00	4.16	9–17
Moderate			11	12.85	11.00	8.38	7–17.3
Severe			0				

*Abbreviations*: Severity *p*-values come from Kruskal-Wallis median test unless (A) marked, those p values come from One-Way ANOVA so far the parameter follows a normal distribution and its n > 30: Δa-d^a^, differences between admission and discharge (Wilcoxons’ test for paired samples p-values); *SD*^b^ Standard deviation, *IQR*^c^ Interquartile range, *IL6*^d^ Interleukin 6, *CRP*^e^ C-reactive protein, *LDH*^f^ Lactate dehydrogenase

At discharge, IL-6, ferritin, D-dimer, LDH, leukocyte, and neutrophil counts remained significantly higher regarding severe cases compared to mild or moderate ones, opposite to lymphocyte percentage (Table 4). CRP values at discharge were close to normal ranges regardless of severity. When comparing laboratory data at discharge with those on admission, an overall return to reference ranges of most parameters was observed, with significantly lower mean values of IL-6, CRP, and LDH, as well as higher mean values of leukocyte counts, neutrophil counts, and lymphocyte counts and percentages. D-dimer and ferritin still remained high or became even higher values upon arrival (Table 4).

**Fig. 2:**
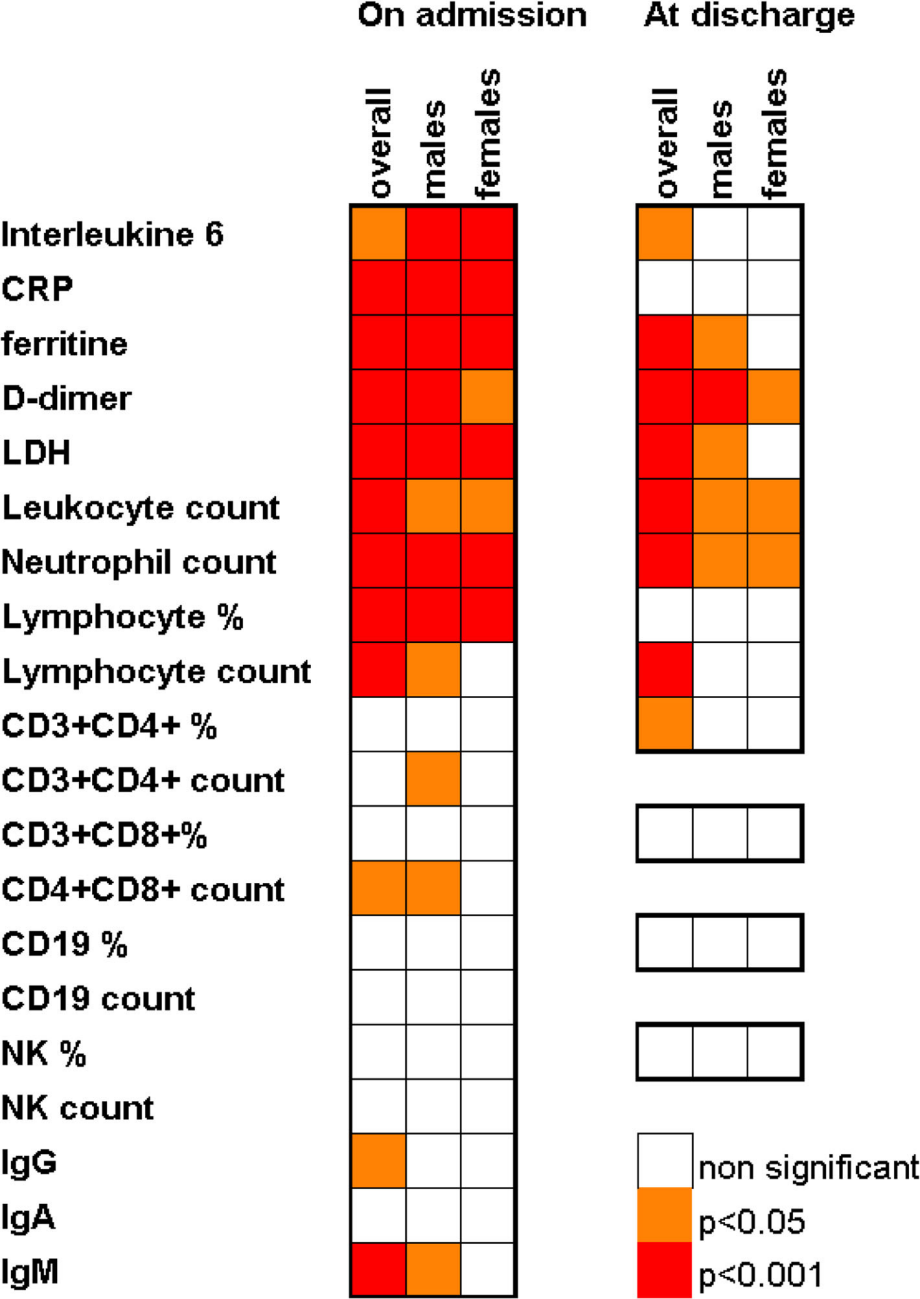
Age related changes of laboratory parameters. Significant associations to severity. Oneway ANOVA (normal n < 30 parameters) and Kruskal Wallis (*n* < 30 or significant Kolmogorov Smirnov test for normal distribution parameters) *p*-values Abbreviations: CRP, C-reactive protein; LDH, lactate dehydrogenase, NK, Natural Killers. IgG, immunoglobulin G; IgA, immunoglobulin A; IgM, immunoglobulin M

**Fig. 3:**
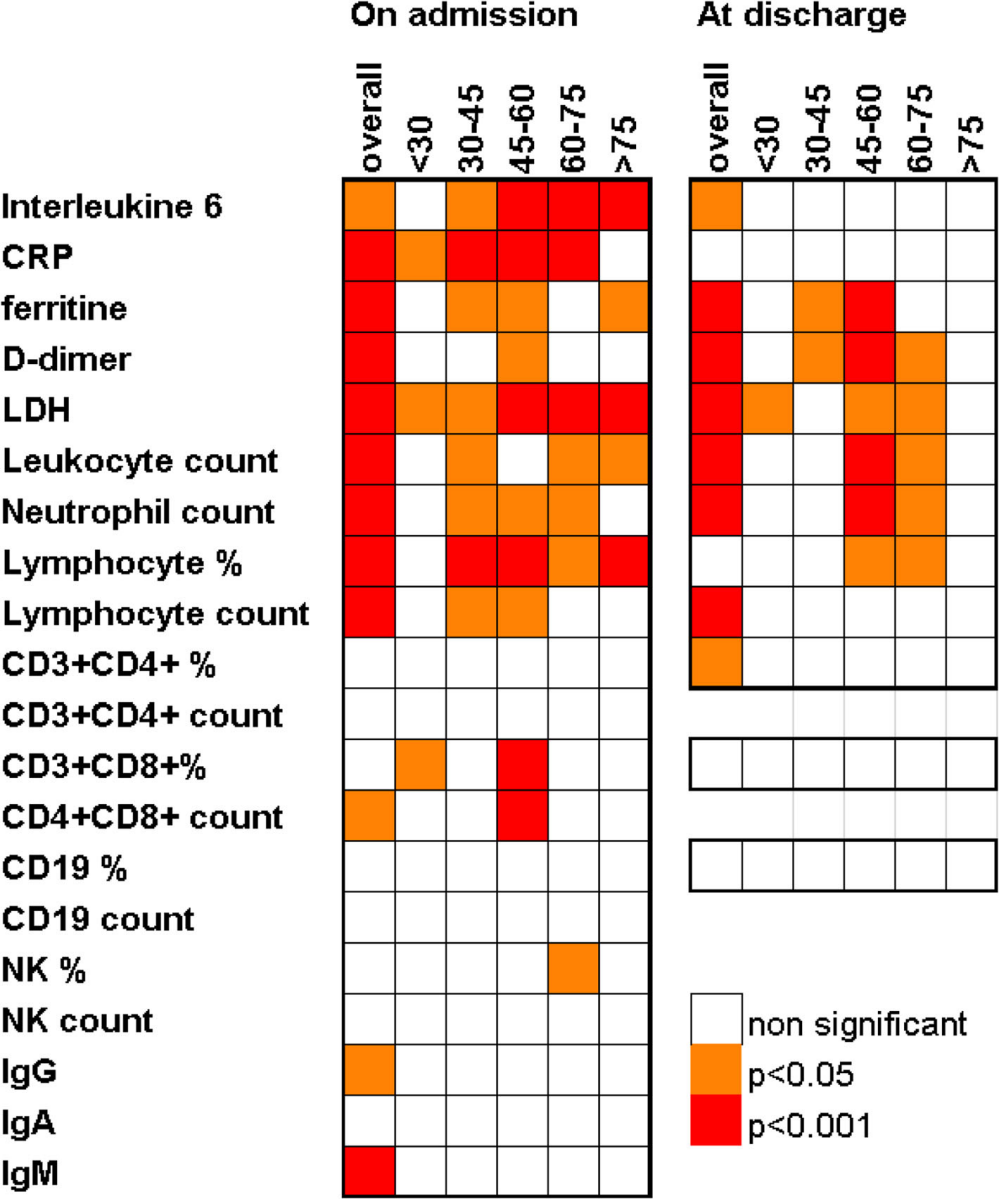
Gender related changes of laboratory parameters. Significant associations to severity. Oneway ANOVA (normal *n* < 30 parameters) and Kruskal Wallis (*n* < 30 or significant Kolmogorov Smirnov test for normal distribution parameters) *p*-values Abbreviations: CRP, C-reactive protein; LDH, lactate dehydrogenase, NK, Natural Killers. IgG, immunoglobulin G; IgA, immunoglobulin A; IgM, immunoglobulin M

Most of the differences in parameter levels amongst the severity groups were the same regardless of age (Fig. 2). It could be evidenced that lymphopenia and increased IL-6 were significant regardless of severity in all age groups but in patients under 30. CD8 population differences (both considering absolute count and percentage) were significant only within the 45–60 group (the largest one). The lymphocyte count decrease, which was seen globally, was only evidenced for 30–45 and 45–60 age ranges. NK percentage was higher in milder cases within older individuals (60–75). Severity-related decreases of IgM (*p* = 0.027), CD4 (p 0.007) and CD8 (p 0.008) lymphocytes were evidenced just in males (Fig. 3).

## Discussion

The COVID-19 pandemic became particularly virulent in Mediterranean countries such as Spain, both in terms of the number of affected people and the fatalities. This is the first report on Spanish COVID-19 inpatients; our aim was to outline illness demographic features, risk factors, and laboratory parameters, in relationship to disease severity. In our series, 27.4% of patients showed a mild course, 42.1% a moderate course, and 30.5% a severe one. Several works analyse severity in COVID-19 inpatients, almost all from Chinese hospitals. Those, including two multicentre studies, can be told to have a low severity profile in which severe cases ranged from 16 to 26% [16–19], except for the study of Zhou et al. [20], where critical cases reached 28%.

It has been reported elsewhere that older patients or those with at least one previous comorbidity have a worse prognosis [16, 19–21]. There is, however, remarkable heterogeneity regarding these studies. The ages of the patients in our series were much higher than those previously published, with an average of 63 years. They can be found in literature, average cohort ages ranging from 36 to 58 years old [16–20, 22–24]. However, Grasselli et al. [25], in a multicentre Italian study focusing on patients admitted to the ICU, reports an average age of 63, similar to ours. In SARS-CoV-2 infection, the number of paediatric patients compared to adults is lower, with milder symptoms and better prognoses [26]. This fact highlights a possible immunosenescence effect on the evolution of the disease [27]. Immunosenescence refers to the age-associated decline of the immune system [28]. Immunosenescence is associated with adaptive immune changes and (less studied) in the innate immune system. These changes within B and T cell compartments do not affect the number of circulating lymphocytes but their repertoire and functionality. Immunosenescence processes include: decreased production of naïve lymphocytes, lymphocyte contracted repertoire, decreased proliferative and functional capacity of effector lymphocytes, increased population of memory lymphocytes, fibrotic changes in lymph node architecture, and cytokine production dysregulation. These phenomena result in a lower vaccination response and a greater infection susceptibility; thus, the infections often evolve more severely. More than 70% of influenza mortality occurs in people over 65 years and the RSV mortality rate within the elderly population is 18% [29]. Knowledge of the mechanisms behind these changes is crucial for vaccine development and for keeping the elderly safe. People over 60 accounts for 11% of the worldwide population and they are expected to reach 22% by 2050 [28]. In Spain, 19.4% of the population is over the age of 65 to date [30]. In our series, 18.15% of the cases were people aged 60 or above.

Possibly due to ageing, frequencies for comorbidities such as hypertension or diabetes were higher in our series than those reported in previous studies [16, 17, 19–24, 31] and the prevalence of diabetes was greater than that recorded in the Spanish adult population (23.7% vs. 13.8%) [32]. Notwithstanding, the prevalence of hypertension mirrored that of the general adult Spanish population (overall, 42.6%; people over 60, 75.4%) [33]. Not only SARS-CoV-2, but most human coronaviruses strike the elderly and individuals with underlying comorbidities harder [34].

As in previous studies, cases were more often males [19, 20, 22, 24]. This fact is noteworthy, considering that men account only for 41.7% of the Spanish population over 50 years old [30] (59.8% in our COVID-19 cohort). Besides, the male gender was found to be associated with severity. In an Italian report of patients admitted to the ICU, up to 80% of the cases were males [25]. This fact would be in line with our observed effect of gender on severity.

Concerning laboratory parameters, our findings were comparable to those reported in previous studies, with elevations of acute phase reactants (CRP, D-dimer, LDH, ferritin) increasing with severity and decreasing when the evolution of patients was favourable [19, 20, 22, 31]. Particularly striking was the change of the CRP, which was almost within its reference range at discharge.

Several publications have focused on immunological markers in COVID-19 [19, 20, 31]. The extensive work of Diao et al. [14] analyses the secretory profile of inflammatory cytokines, lymphocyte populations, and their relationship to disease severity in 499 patients. The authors find an increase in pro-inflammatory cytokines inversely correlated to T-lymphocyte populations. This immune profile was also related to the severity of the disease. CD4, CD8, and IL-6 are reported to covariate at least in mild cases [35]. Data in our series would ratify their findings, therefore, an increase in IL-6 and a decrease in both total lymphocytes and lymphocyte populations could be seen. Once again, these changes were greater the more severe the condition. In our cohort, all lymphocyte subset counts CD4, CD8, CD19, and NK were below reference ranges upon arrival and were strongly decreased in severe cases, despite the differences being only significant for the CD8 population regarding overall data. Lymphopenia has been described in other infectious contexts such as sepsis, HIV, SARS, and MERS infections, [36, 37]. The underlying cause of lymphopenia in severe cases of COVID-19 is still unknown and several mechanisms have been proposed to explain it. Some of these hypotheses are apoptosis of T lymphocytes [38], an IL-induced pyroptosis-1β [38], a direct cytopathic virus action on T lymphocyte [39], a bone marrow suppression due to cytokine storm (similar to that in sepsis), or pulmonary sequestration by bilateral pneumonia [40]. Quantitative alterations in the effectors of the immune system such as lymphopenia and increased levels of IL-6, together with possible qualitative alterations associated with the ageing of the immune system, could act synergistically, causing a more serious condition.

Since the seminal publication of Lei Fang et al. on the possible involvement of renin-angiotensin system blockers in SARS-CoV-2 infection [10] just 3 months ago, there has been a lot of controversy about it. No sooner had the scientific community realised its foreseeable impact, they began to take sides both for and against the hypothesis [41–44]. ACE2 molecules are the door used by SARS-CoV-2 to enter the cell [45]. RASBs indirectly increase the expression and secretion to the extracellular medium of ACE2 in various cell types, including airway alveolar epithelial cells [41]. RASBs might therefore facilitate the entrance of the virus or prevent it [42]. Additionally, the expression of ACE2 is associated with positive effects on lung homeostasis, which could be beneficial for tissue recovery from the damage caused by the SARS-CoV-2 infection [11, 42]. ACE2 expression is reported to be related to age and sex. It is high in children and would be high in young women, decreasing with ageing, and correlated negatively with chronic disease comorbidities such as hypertension [46]. ACE2 levels will inversely correlate COVID-19 severity and poor outcomes. Most literature for or against the role of the use of RASB consists mainly of theoretical positioning based on the knowledge of the physiological properties of these drugs. There is a limited number of original studies analysing the RASB intake effect on COVID-19. In our series, 293 patients were hypertensive. From these, 265 had records of being on anti-hypertensive drugs in their clinical history; of the latest, 209 were on RASB (78.86%; this feature is similar to the reported overall intake of these drugs by the Spanish hypertensive population [33]. No differences in severity concerning the use of RASB were found. Notwithstanding, when RASB were separately analysed, ARBs were found to be associated with a worse course of the disease (p 0.004) and ACEI with a better evolution (p 0.046). A lack of association between the use of RASB and severity has been previously reported by several authors. Tedeschi et al. [47], to elucidate whether RASB treatment had an impact on COVID-19 mortality, analyse 311 hypertensive patients hospitalized in 10 Italian centres. A multivariate Cox regression analysis of intra-hospital mortality shows that the use of RASBs is not associated with outcome. A large population-based case-control study by Mancia et al. [48] including 6272 hypertensive patients with COVID-19 disease has just been published, where the RASBs intake effect on COVID-19 is analysed. They conclude that neither susceptibility nor disease severity is associated with RASB intake. Even more, Chen et al. [31] report about 113 hypertensive patients, 33 (29.2%) of them were on RASB treatment, 87.9% of whom had moderate disease, and 12.2% a severe or critical COVID-19. Nevertheless, in contrast with our results, when Mancia et al. separately address the ACEI and ARB effects on the severity, they conclude that neither ACEI nor ARB show an independent association with COVID-19 severity. This difference might be due to the structure of the cohorts. It shall be noticed that all patients in our series were cases hospitalized. Even those here so categorized as having a mild course required hospitalization. Another main difference is related to the criteria used to define when a patient was on therapy with antihypertensive drugs. In our series, any intakes by disease onset were considered, whereas, in the mentioned study, even the whole preceding year was considered. Hence, these differences indicate that further studies to clarify the possible roles of various types of RASB in COVID-19 prognosis are warranted.

The present study has two major limitations. The first one is derived from its retrospective design. As we are reporting on the very first cases of the disease in Spain, several immunological parameters and risk factors of interest were not systematically tested or recorded into medical history. The other restraint is the short follow-up period of patients, which limits the possibility of having a complete follow up of those who were still in hospital by the data collection deadline.

## Conclusions

Age has emerged as a crucial factor in our series. Age is also one of the major determinants for all other COVID-19 risk comorbidities, such as hypertension, diabetes, or dyslipidaemia. Angiotensin-converting enzyme inhibitors used by hypertensive patients would have a protective effect against COVID-19 severest forms, opposite to angiotensin II receptor blockers. Our patients are older and develop therefore a severe COVID-19 more often than the previously reported cohorts. Immunosenescence might be a suitable explanation for the immune overwhelming observed in the severest cases. Regarding not only our series but other ones around the world, the effectors of the immune system are hampered as severity increases. Adaptive immunity has been suggested to be disabled by SARS-CoV-2. That feature has been referred to as immunity exhausted. This exhaustion may be coupled with a huge ineffective and almost deleterious innate response.

Further studies on the immune system status in SARS-CoV-2 infected patients should be carried out to support the immunosenescence hypothesis as well as deeper analyses on RASB intake. Our data highlight that the elderly are at a special risk of COVID-19 and should therefore be monitored closely by public health services.

## Methods

### Aim, design, and setting of the study

This study aimed to reveal risk factors regarding severity by outlining, within the shortest possible time, a reliable snapshot of the demographic and clinical characteristics of COVID-19 patients admitted to Spanish hospitals along the first month of the pandemic.

### Participants

Our multicentre cohort consisted of the first consecutive set of SARS-CoV-2 infected inpatients, confirmed by a positive PCR test, during the second half of March 2020. Cases were tracked for a three-week follow-up period from admission to discharge. A minimum sample size of 20 patients was considered for every hospital. A total of 642 medical records of individuals over 18 years old, from 19 Spanish hospitals were initially reviewed. After data quality assessment, 584 patients were included in the analyses. Participants were stratified into three severity groups before analysis according to the following clinical criteria:

- Mild: individuals whose clinical symptoms were mild with no abnormal radiological findings
- Moderate: cases with confirmed, non-severe pneumonia
- Severe: those so considered by the physician in charge or meeting at least one of the following criteria: acute respiratory distress, shock, admission to the intensive care unit (ICU). Any “exitus” was as well classified as a severe case.

### Data collection

All data were extracted from electronic medical records. The collection form included demographic, epidemiological and clinical data: age, sex, diabetes mellitus (DM), dyslipidaemia, hypertension (HTA), renin-angiotensin system blocker intake (RASB), COVID-19 severity, time from symptom onset to diagnosis, laboratory data on admission and discharge, treatment, and outcome. At the end of data collection, some patients were still in the hospital. In these cases, laboratory data at discharge could not be provided.

### Laboratory data

Requested laboratory markers were extracted from medical records on admission and at discharge. Routine blood examinations included leukocyte, neutrophil, and lymphocyte count (cells*10^3/μL) and lymphocyte percentage. Serum biochemical tests recorded were ferritin (μg/L), lactate dehydrogenase (LDH, U/L), C- reactive protein (CRP, mg/L), and D-dimer (μg/L). Immunological tests recorded were interleukin-6 (IL-6, pg/mL), Lymphocyte population count (cells*10^3/μL), and the percentage by flow cytometry, immunoglobulins IgG, IgA, and IgM (mg/dL).

### Statistical analysis

Demographic and clinical characteristics of patients were expressed as their mean and standard deviation (SD); when not adjusting to a normal distribution, the median was used to represent non-parametrical data for continuous variables and frequency distributions are reported for categorical variables. Age was analysed both, as a continuous and categorical variable, recoded then into 5 groups: < 30, 30–45, 45–60, 60–75, and > 75.

Continuous variables: 1. Normality testing: Kolmogorov-Smirnov test was performed on each continuous variable with more than 30 valid cases to contrast their normal distribution. Any variable with less than 30 valid cases was considered non-parametric for further hypothesis tests. 2. The difference of means (normal variables): To analyse the overall differences between the three groups: mild, moderate, and severe, ANOVA was tested on variables with normal distribution and *n* > 30 (age, percentage and CD4 lymphocyte count, percentage of CD8 lymphocytes, percentage of CD19 lymphocytes and percentage of NK). 3. The difference of medians: To analyse severity relationships of non-parametric or *n* < 30 variables, a Kruskal-Wallis test was used. 4. Changes along COVID-19: To compare values of recurrent parameters measured in the same case on admission and at discharge, the Wilcoxon test for paired data was performed. Categorical variables: To contrast the “Ho” of independence within categorical variables, Pearson’s Chi-square and Fisher’s exact test were used.

## Data Availability

All collected data, including fully anonymized participant data, are available to others. Available information includes fully anonymized participant data and data dictionary. Related documents are available from the date of publications henceforth: study protocol, statistical analysis, and approval of the ethical board. These documents are available from the date of publications henceforth at email address cmartinalo@saludcastillayleon.es or aurora.jurado.sspa@juntadeandalucia.es Data will be shared after approval of proposals by the Valladolid Este Ethical Committee.

## Abbreviations

ACE2: Angiotensin-converting enzyme 2
ACEI: Angiotensin-converting enzyme inhibitors
ARB: Angiotensin II receptor blockers
RASB: Renin-angiotensin system blockers
LDH: Lactate dehydrogenase
CRP: C- reactive protein
IL-6: Interleukin-6
IgG: Immunoglobulin G
IgA: Immunoglobulin A
IgM: Immunoglobulin M
PCR: Polymerase chain reaction
SD: Standard deviation
IQR: Interquartile range
NK: Natural Killers
